# The benefits of using confocal laser endomicroscopy in the diagnosis of gastric cancer and precancerous lesions: a case report

**DOI:** 10.3389/fonc.2025.1649985

**Published:** 2025-10-06

**Authors:** Xiaoting Peng, Ya-Ping Zheng, Yan Zhang

**Affiliations:** Department of Gastroenterology, Affiliated Puren Hospital of Wuhan University of Science and Technology, Wuhan, Hubei, China

**Keywords:** confocal laser endomicroscopy, early diagnosis, gastrointestinal cancer, chronic gastritis, optical biopsy

## Abstract

**Background:**

Cases of early gastrointestinal cancers (EGC) are often identified as advanced stages due to the lack of typical clinical manifestations, leading to missed or delayed diagnoses. Confocal laser endomicroscopy (CLE), a novel microscopic imaging technique, enables real-time *in vivo* histological examination during endoscopy, providing a valuable tool for the early detection of EGC.

**Intervention:**

A 62-year-old woman with a gastric antral lesion, considered a polyp or early cancer, underwent CLE during gastroscopy to refine and confirm the diagnosis.

**Findings:**

CLE imaging showed mild irregularities in the glandular architecture, thickening of the mucosal margins, the presence of pleated structures, dilated glandular openings, increased vascularity, and mild fluorescein leakage, all consistent with mild to moderate inflammatory changes. No features suggestive of malignant lesions were revealed.

**Conclusion:**

CLE may play a pivotal role in the early differentiation of benign and malignant gastrointestinal lesions. While not yet a replacement for histopathology, CLE demonstrates significant clinical utility and potential for improving the accuracy of early cancer detection and long-term monitoring.

## 1 Introduction

Recently, gastric cancer (GC) has emerged as a major global health concern, with rising incidence and mortality rates, now ranking the fourth among all cancers (1). The pathogenesis of GC involves a multistep progression from chronic gastritis, chronic atrophic gastritis (AG), gastrointestinal metaplasia (GIM), and gastric intraepithelial neoplasia (GIN), representing key precancerous stages, to early gastric cancer (EGC) (2). Early detection of GC and its precursors is critical for improving treatment outcomes and prognosis. Traditional white-light endoscopy (WLE) is limited in detecting subtle lesions, as it primarily visualizes mucosal and submucosal changes, such as atrophy or intestinal metaplasia (3). WLE’s tumor detection miss rate remains high, ranging from 4.6% to 25.8% (4). In contrast, confocal laser endomicroscopy (CLE), a novel imaging technique, enables real-time *in vivo* histological assessment during endoscopy, termed “optical biopsy” (5). Its principle relies on blue laser illumination and fluorescence detection, with only focal plane light passing through a pinhole to enhance resolution and contrast (6). CLE also provides 1000× magnification, allowing cellular or subcellular visualization of mucosal structures.The Miami Classification system is widely adopted in clinical practice to standardize the interpretation of CLE imaging and establish diagnostic criteria for gastrointestinal mucosal alterations. This framework categorizes mucosal changes into four distinct classes based on three key microstructural features: (1) glandular architecture, (2) cellular morphology, and (3) microvascular patterns. Within this classification, early gastric cancer (EGC) is defined by a diagnostic triad: completely disorganized epithelium, heterogeneous fluorescein leakage, and dark irregular epithelial cells with loss of polarity (7) Here, CLE was employed for intraoperative diagnosis in a female with gastrointestinal symptoms, and demonstrated its clinical use for real-time lesion characterization and improved diagnostic precision.

## 2 Case presentation

The diagnostic trajectory is summarized in **Table 1**.

**Table 1:** Timeline of diagnostic interventions and key findings in the present case.

Date	Symptoms	Investigations	Key findings
04 June 2024	Heartburn, acid reflux	Gastroscopy + Endoscopic ultrasonography (EUS)	1. Bulge on lesser curvature of gastric antrum 2. Slightly hyperechoic mucosal lesion in gastric antrum
26 December 2024	Intermittent epigastric pain	Repeat gastroscopy	Persistent antral bulge
28 December 2024	Persistent pain	Confocal laser endomicroscopy	Mild-to-moderate inflammatory changes: • Mildly irregular glandular architecture • Thickened mucosal margins • Minimal fluorescein leakage
31 December 2024	–	Histopathological examination	Moderate mucosal inflammation (Activity+++)with reactive glandular changes

### 2.1 Clinical history

A 62-year-old female was admitted on December 25, 2024, with a six-month history of a suspected gastric space-occupying lesion. Six months earlier, the patient underwent a painless gastroscopy and endoscopic ultrasound, which revealed antral protrusion along with mildly hyperechoic mucosal lesions suggestive of polyps or early-stage cancer. Symptoms were temporarily attenuated after self-administered medication. However, 4 weeks later, progressive epigastric pain, postprandial exacerbation, nocturnal discomfort, nausea, occasional vomiting, dysphagia, acid reflux, and fatigue with a 5 kg weight loss were reported.

### 2.2 Physical examination and past medical history

Physical examination revealed no significant abnormalities. The patient had a history of *Helicobacter pylori* (Hp) infection, erosive gastritis, stable angina, coronary artery disease, and tricuspid insufficiency.

### 2.3 Diagnostic and therapeutic course

**Figure 1:**
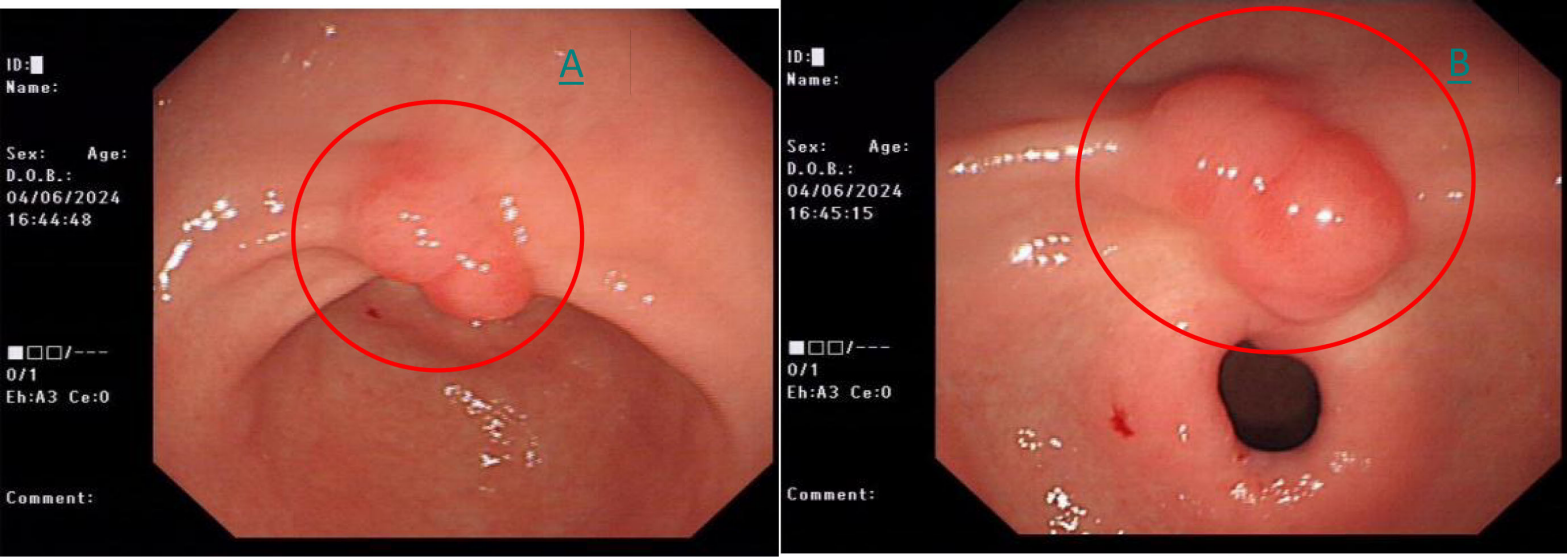
**(A)** White-light endoscopy shows a 1.2*0.8cm red and elevated lesion on the lesser curvature of stomach (red circle). **(B)** Close-up picture of the same lesion in white light (red circle).

All gastroscopy and confocal examinations of the patient were conducted with informed consent, in compliance with ethical requirements. During the CLE examination, the patient did not show any discomfort. The initial gastroscopy on June 4, 2024 revealed a 1.2 × 0.8 cm reddish, smooth-surfaced bulge on the lesser curvature of the gastric antrum (**Figure 1**).

**Figure 2:**
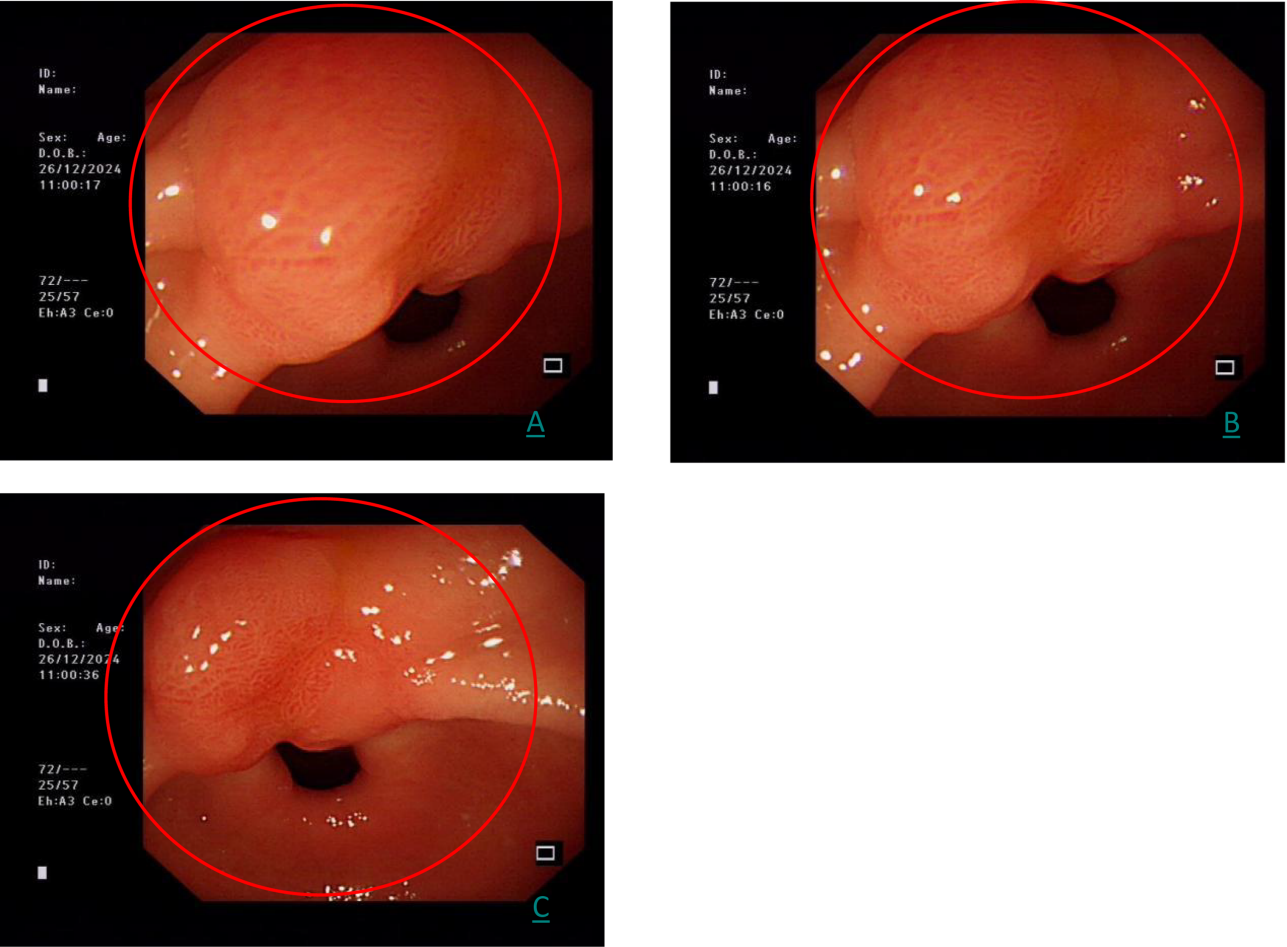
**(A–C)** represents the three images of the same gastric antrum lesion at different angles by white light endoscopy (red circle).

A follow-up gastroscopy on December 26, 2024 showed the same lesion, which is consistent with the initial examination (**Figure 2**).

**Figure 3:**
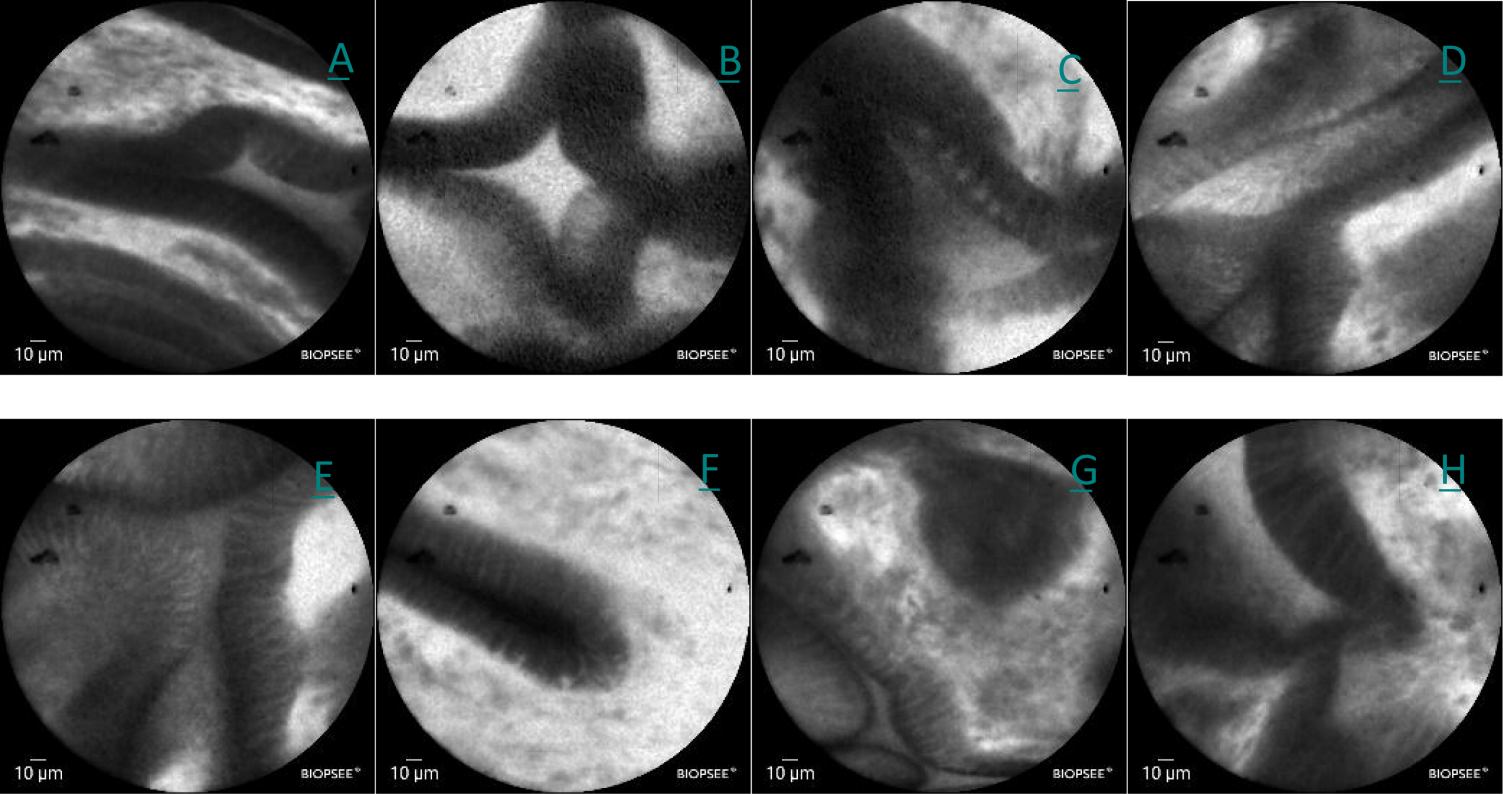
**(A–H)** Confocal images of lesions in the lesser curvature of the stomach in white light, showing a mild irregularity in the arrangement the glands, slight thickening of the edges, folding, dilation of the orifices, a slight increase in the number of vessels, and a small amount of flucein sodium leakage.

A CLE examination was performed on December 28, 2024. After a negative fluorescein sodium allergy test, intravenous fluorescein sodium was administered. CLE imaging using a BIOPSEE^®^ probe revealed mildly irregular glandular architecture, thickened mucosal margins, folded structures, dilated glandular openings, increased vascularity, and minimal fluorescein leakage, consistent with mild-to-moderate inflammatory changes (**Figure 3**).

**Figure 4:**
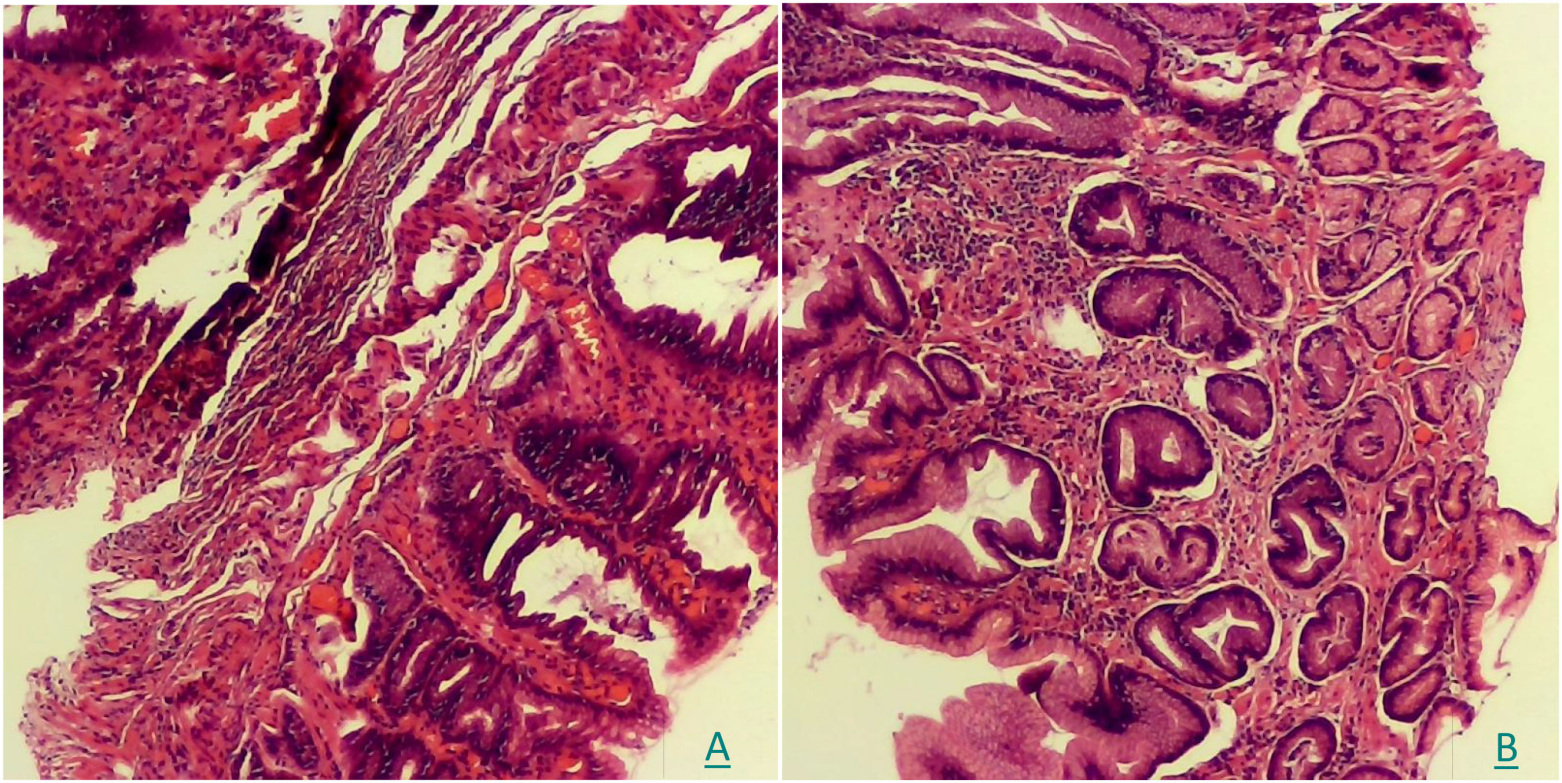
**(A, B)** The pathological examination of HE staining showed moderate mucosal inflammation (activity+++) with hyperplastic polypoid changes and reactive glandular alterations.

A histopathological analysis was conducted using the biopsy samples when the CLE was performed. The examination confirmed moderate mucosal inflammation (activity+++) with hyperplastic polypoid changes and reactive glandular alterations (**Figure 4**).

### 2.4 Final diagnosis

Integrated findings from gastroscopy, CLE, and histopathology confirmed gastric antral inflammatory lesions without evidence of malignancy.

### 2.5 Follow-up

At one-month post-discharge, telephone follow-up revealed resolution of gastrointestinal symptoms with no reported epigastric discomfort. The patient exhibited favorable recovery indicators: normal mental status, adequate nutritional intake, and stable sleep patterns. Notably, she expressed strong preference for CLE over repeated biopsies, citing *“significantly reduced post-procedural discomfort”*. Furthermore, she reported high satisfaction with the diagnostic efficiency (*“The real-time diagnosis effectively alleviated my anxiety during the waiting period for histopathology results”*). Written informed consent for experience publication was reconfirmed.

## 3 Discussion

Chronic gastritis, characterized by persistent inflammation of the gastric mucosa, is frequently associated with Hp infection, one of its most common etiological factors. Patients typically present with epigastric discomfort or a burning sensation. Left untreated, chronic gastritis carries a risk of malignant transformation, emphasizing the importance of early and accurate diagnosis, as well as the timely pharmacological intervention to control disease progression and improve patient prognosis. CLE significantly altered clinical management in this case. Initial gastroscopic and endosonographic findings identified a slightly hyperechoic mucosal lesion in the gastric antrum, yet failed to definitively characterize its nature. This diagnostic uncertainty necessitated plans for invasive procedures. Critically, CLE provided real-time intraoperative diagnosis of benign inflammation (later corroborated by histopathology), thereby enabling a shift to conservative pharmacotherapy and avoiding unnecessary surgery. Notably, CLE delivers immediate pathological insights days before conventional histopathology, offering clinicians accelerated therapeutic decision-making. Additionally, CLE enables Hp visualization using acriflavine staining, demonstrating 93% sensitivity and 86% specificity for diagnosing Hp-associated gastritis (8).

CLE requires fluorescent agents, typically administered intravenously, with fluorescein sodium being the most common. This agent highlights cellular and subcellular details without nuclear staining. CLE systems are categorized into three types: endoscope-based CLE (eCLE), probe-based CLE (pCLE), and needle-based CLE (nCLE). eCLE integrates a confocal microscope into the endoscope’s distal end but is rarely used due to poor maneuverability. pCLE employs a microprobe inserted through the biopsy channel, though its scanning depth is fixed. nCLE, a miniaturized version of pCLE, allows microscopic imaging of deeper mucosal or visceral tissues during endoscopic ultrasound (EUS) via a 19G needle, overcoming pCLE’s limitation to superficial lesions. Currently, nCLE is the most widely used in clinical practice (9).Compared with conventional biopsy, the combination of conventional biopsy or *in vivo* pCLE demonstrated significantly higher sensitivity (96.9% vs. 75.0%) (10).This supports pCLE as a valuable adjunct to traditional biopsy, enabling targeted sampling or biopsy replacement in endoscopically suspicious lesions to mitigate tissue damage from repeated procedures.As summarized in **Table 2**, CLE exhibits excellent diagnostic performance for gastric precancerous lesions:For high-grade intraepithelial neoplasia (HGIN), CLE achieved a sensitivity of 95.8% and specificity of 97.2% using the Li criterion (11);FICE-guided pCLE demonstrated 95.0% sensitivity and 94.6% specificity in detecting GIM (12).Critically, CLE combined with targeted biopsy (e.g., the Guo criterion (13)) optimizes diagnostic workflows by reducing sampling requirements.Further comparisons with endoscopic techniques (**Table 3**) reveal:CLE significantly outperformed WLE in early gastric cancer (EGC) diagnosis (97% vs. 72%, *p* < 0.05) (14);For AG, CLE provided higher specificity than autofluorescence imaging (AFI) (84.7% vs. 69.5%, *p* < 0.05) (15).Thus, CLE allows for biopsy reduction while maintaining diagnostic accuracy, demonstrating its clinical utility in risk stratification.CLE offers significant advantages: it reduces biopsy requirements, enhances diagnostic sensitivity, and minimizes complications such as mucosal injury, infection, and bleeding. These attributes make it ideal for long-term monitoring of early gastric cancer (6). It also enables rapid intraoperative assessment of epithelial cells, glandular morphology, and microvascular structures, shortening diagnostic timelines and improving tumor margin evaluation (16, 17).However, CLE has limitations: its narrow field of view restricts examination of the entire gastric cavity, respiratory movements can cause instability, and fluorescein’s inability to stain nuclei limits diagnosis to structural atypia (18). Additionally, its shallow tissue penetration hinders visualization of deeper structures, and high costs and specialized training requirements limit its widespread adoption (19). Despite these limitations, CLE remains a promising imaging technology for detecting gastrointestinal malignancies.

**Table 2:** Diagnostic performance of CLE for gastric precancerous lesions.

Lesion type	Diagnostic criteria	Sensitivity (%)	Specificity (%)	Reference standard	Technique combination
AG	New Criterion (13)	90.3	78.8	Histopathology	pCLE
	Li New Classification (11)	92.3	99.3	Histopathology	pCLE
GIM	FICE-guided (12)	95.0	94.6	Histopathology	FICE + pCLE
	Guo Criterion (20)	91.7	96.8	Histopathology	CLE + Targeted Biopsy
LGIN	FICE-guided (12)	87.5	98.0	Histopathology	FICE + pCLE
	Li Criterion (21)	85.3	87.5	Histopathology	pCLE
HGIN	Li Criterion (21)	95.8	97.2	Histopathology	pCLE

**Table 3:** Comparative performance of CLE versus other endoscopic techniques.

Lesion type	Lesion type	CLE performance	Comparative technique performance	Statistical significance
White-light endoscopy (WLE)	GIM detection	65.7% (targeted biopsy)	15.7% (random biopsy)	*p* < 0.001
	EGC diagnosis	97% (diagnostic yield)	72% (diagnostic yield)	*p* < 0.05
Magnifying endoscopy with NBI (ME-NBI)	Cancer/HGIN	Sensitivity 91.7%, Specificity 95.5%	Sensitivity 90.0%, Specificity 93.5%	NS (*p* > 0.05)
	EGC margin	Accuracy 91.7%	Accuracy 69.4%	*p* < 0.05
Autofluorescence imaging (AFI)	AG diagnosis	Sensitivity 90.9%, Specificity 84.7%	Sensitivity 68.2%, Specificity 69.5%	*p* < 0.05
Chromoendoscopy (CE)	AG diagnosis	Sensitivity 92.3%, Specificity 86.2%	Sensitivity 83.9%, Specificity 78.9%	*p* < 0.05
Virtual chromoendoscopy (FICE)	GIM detection	Specificity 94.6% (combined)	Specificity 79.2% (alone)	*p* = 0.004

*p<0.05 vs. comparison group.

## 4 Conclusion

CLE represents a transformative advancement in endoscopic imaging, offering real-time histological evaluation for early detection of precancerous and malignant gastric lesions. While complementary to traditional histopathology, its integration into clinical practice holds promise for improving diagnostic accuracy and patient outcomes. Future research should focus on overcoming technical limitations and expanding its applications in gastrointestinal oncology.

## Data Availability

The original contributions presented in the study are included in the article/supplementary material. Further inquiries can be directed to the corresponding author.
